# Combination of exercise and GLP-1 receptor agonist treatment reduces severity of metabolic syndrome, abdominal obesity, and inflammation: a randomized controlled trial

**DOI:** 10.1186/s12933-023-01765-z

**Published:** 2023-02-25

**Authors:** Rasmus M. Sandsdal, Christian R. Juhl, Simon B. K. Jensen, Julie R. Lundgren, Charlotte Janus, Martin B. Blond, Mads Rosenkilde, Adrian F. Bogh, Lasse Gliemann, Jens-Erik B. Jensen, Charalambos Antoniades, Bente M. Stallknecht, Jens J. Holst, Sten Madsbad, Signe S. Torekov

**Affiliations:** 1 Department of Biomedical Sciences, Faculty of Health and Medical Sciences, University of Copenhagen, Blegdamsvej 3B, DK-2200 Copenhagen N, Denmark; 2 Steno Diabetes Center Copenhagen, Gentofte, Denmark; 3 The August Krogh Section for Human Physiology, Department of Nutrition, Exercise and Sports, University of Copenhagen, Copenhagen, Denmark; 4 Department of Clinical Medicine, University of Copenhagen, Copenhagen, Denmark; 5 Department of Endocrinology, Hvidovre University Hospital, Copenhagen, Denmark; 6 Division of Cardiovascular Medicine, Radcliffe Department of Medicine, University of Oxford, Oxford, UK; 7 Novo Nordisk Foundation Center for Basic Metabolic Research, University of Copenhagen, Copenhagen, Denmark

**Keywords:** Metabolic syndrome, Obesity, Inflammation, Cardiometabolic risk, GLP-1, Exercise, Randomized clinical trial

## Abstract

**Background:**

Identifying and reducing cardiometabolic risks driven by obesity remains a healthcare challenge. The metabolic syndrome is associated with abdominal obesity and inflammation and is predictive of long-term risk of developing type 2 diabetes and cardiovascular disease in otherwise healthy individuals living with obesity. Therefore, we investigated the effects of adherent exercise, a glucagon-like peptide 1 receptor agonist (GLP-1 RA), or the combination on severity of metabolic syndrome, abdominal obesity, and inflammation following weight loss.

**Methods:**

This was a randomized, double-blinded, placebo-controlled trial. During an 8-week low-calorie diet (800 kcal/day), 195 adults with obesity and without diabetes lost 12% in body weight. Participants were then evenly randomized to four arms of one-year treatment with: *placebo*, moderate-to-vigorous *exercise* (minimum of 150 min/week of moderate-intensity or 75 min/week of vigorous-intensity aerobic physical activity or an equivalent combination of both), the GLP-1 RA *liraglutide* 3.0 mg/day, or a *combination* (exercise + liraglutide). A total of 166 participants completed the trial. We assessed the prespecified secondary outcome metabolic syndrome severity z-score (MetS-Z), abdominal obesity (estimated as android fat via dual-energy X-ray absorptiometry), and inflammation marker high-sensitivity C-reactive protein (hsCRP). Statistical analysis was performed on 130 participants adherent to the study interventions (per-protocol population) using a mixed linear model.

**Results:**

The diet-induced weight loss decreased the severity of MetS-Z from 0.57 to 0.06, which was maintained in the placebo and exercise groups after one year. MetS-Z was further decreased by liraglutide (− 0.37, 95% CI − 0.58 to − 0.16, P < 0.001) and the combination treatment (− 0.48, 95% CI − 0.70 to − 0.25, P < 0.001) compared to placebo. Abdominal fat percentage decreased by 2.6, 2.8, and 6.1 percentage points in the exercise, liraglutide, and combination groups compared to placebo, respectively, and hsCRP decreased only in the combination group compared with placebo (by 43%, P = 0.03).

**Conclusion:**

The combination of adherent exercise and liraglutide treatment reduced metabolic syndrome severity, abdominal obesity, and inflammation and may therefore reduce cardiometabolic risk more than the individual treatments.

*Trial registration* EudraCT number: 2015-005585-32, ClinicalTrials.gov: NCT04122716

**Supplementary Information:**

The online version contains supplementary material available at 10.1186/s12933-023-01765-z.

## Background

Identifying and reducing cardiometabolic risks driven by obesity remains a major healthcare challenge [1]. Metabolic syndrome (MetS) is associated with an increased risk of cardiovascular disease, type 2 diabetes, and all-cause mortality [2, 3]. Abdominal obesity is associated with low-grade inflammation and has been proposed as a driver for metabolic syndrome [4]. Body weight loss may improve the factors of MetS [5]; however, weight loss-induced improvements have proven difficult to maintain since substantial weight regain often occurs within the first year [6]. Therefore, investigations of treatment strategies that can maintain, or even reduce, metabolic syndrome, abdominal fat, and low-grade inflammation in currently healthy persons with obesity to prevent future cardiometabolic disease are warranted [7].

MetS denotes a cluster of common risk factors and was intended as an early measure for cardiometabolic disease risk [8]. However, the dichotomous design of MetS has its limitations, and it is debated whether different definitions of MetS add predictive value when adjusted for its individual factors [9, 10]. The newer metabolic syndrome severity z-score (MetS-Z) combines weighted contributions of all MetS factors into a single continuous measure [11]. Studies have shown that individuals within the fourth quartile of MetS-Z scores (> 0.675) had a hazard ratio (HR) of 5.1 for coronary heart disease with more than 11 years of follow-up [12, 13] and 17.4 for future diabetes with a median follow-up of 8 years compared to those from the first quartile of MetS-Z scores. However, MetS-Z has not been investigated in randomized clinical trials comparing treatments during weight loss maintenance in people at risk of future cardiometabolic disease.

High-sensitivity C-reactive protein (hsCRP) is an established biomarker of inflammation [14] and is commonly elevated in persons with obesity [15]. The relationship between hsCRP and the risk of cardiovascular disease is well documented; hsCRP levels of < 1 mg/L, 1–3 mg/L, or > 3 mg/L can be used to classify the risk of cardiovascular risk as low, intermediate, or high (in combination with traditional cardiovascular risk factors) [14, 15].

Exercise and glucagon-like peptide–1 receptor agonists (GLP-1 RAs) may be different strategies in the primary and secondary prevention of MetS, abdominal fat, and inflammation [7].

A meta-analysis has shown that moderate-to-vigorous aerobic exercise for at least 12 weeks can improve the factors of MetS [16], and a study of self-reported physical activity has shown that exercise was associated with reduced inflammation markers in 10 years of follow-up [17]. The potential anti-inflammatory effects of exercise might, in part, be due to reduced visceral fat independent of total body weight loss [4, 18]. However, determining the effects of exercise interventions is often complicated by high study heterogeneity and, importantly, varying adherence to intervention protocols [19]. Thus, studies that assess the effects of exercise adherent to intervention protocols are limited.

The GLP-1 RA, liraglutide, approved for obesity therapy, induces weight loss and improves glycemic control and cardiovascular risk factors (e.g., lipid profile and blood pressure) [20, 21]. GLP-1 RAs are also suggested to lower inflammation due to direct anti-inflammatory effects on various tissues and immune cells and partly because of the weight loss seen with GLP-1 RA treatment [22, 23].

We recently showed that a diet-induced 12% weight loss was maintained after one year with either exercise or liraglutide treatment. Combining the two treatments led to additional weight loss, while the placebo group regained body weight [24]. In the present study, we investigated the effects of actually performed moderate-to-vigorous exercise, liraglutide 3.0 mg/day, or the combination of exercise and liraglutide on MetS-Z, abdominal obesity, and the inflammation marker hsCRP in a one-year maintenance period following a diet-induced weight loss.

## Materials and methods

### Study design

This study is based on a randomized, double-blind (regarding liraglutide treatment), placebo-controlled trial (S-LiTE Randomized trial) conducted at Hvidovre Hospital and the University of Copenhagen, Denmark, from August 2016 to November 2019 (EudraCT number, 2015-005585-32; ClinicalTrials.gov number, NCT04122716) [25]. Details on methods and results regarding the primary endpoint (change in body weight) and a secondary endpoint (change in total body fat percentage) have previously been published [24]. This study presents the analysis of the prespecified secondary endpoint MetS-Z, and android fat and hsCRP.

Included participants were asked to complete a low-calorie diet for eight weeks before being randomized to either exercise, pharmacological treatment with liraglutide, the combination of exercise and liraglutide, or placebo for one year. All participants attended 12 individual consultations to support weight loss maintenance after randomization. These consultations included measurements of body weight and dietary support in compliance with the Danish Authorities’ dietary recommendations [25]. A full description of weight loss maintenance support during the trial, including dietary advice, can be found here [24].

The trial was approved by the Committee of Health Research Ethics and the Danish Medicines Agency and was conducted according to the Declaration of Helsinki and Good Clinical Practice guidelines. Participants provided written informed consent before inclusion. Investigators, assessors, and participants were blinded to study medication. Unblinding was done after the statistical analysis of the primary and secondary endpoints [24].

### Participants

Recruited participants were adults living with obesity (18–65 years of age, BMI 32–43 kg/m^2^). Major exclusion criteria were any known serious chronic illness, including type 1 or 2 diabetes (see the full list of exclusion criteria in the protocol article of the trial [25]). A total of 215 participants were enrolled in the trial, of which 195 completed the low-calorie diet and were randomized (week 0) in a 1:1:1:1 ratio stratified by sex (male/female) and age (< / ≥ 40 years) to placebo (n = 49); exercise (n = 48); liraglutide (n = 49); liraglutide and exercise (n = 49) for one year [25]. Based on a randomization list (provided by Novo Nordisk), a study nurse performed the allocation of participants to treatment.

The per-protocol population was defined as participants that met the pre-defined criteria of performing at least 75% of WHO recommendations on physical activity (150 min/week of moderate-intensity, or 75 min/week of vigorous-intensity aerobic physical activity, or an equivalent combination of both) and having administered 2.4 or 3.0 mg/day of liraglutide/placebo for at least 75% of the intervention period [25, 26]. The intention-to-treat population was all 195 randomized participants regardless of adherence to the study interventions. See CONSORT diagram for study flow, Additional file 1: Fig. S1.

### Interventions

Participants followed a low-calorie diet of 800 kcal/day (meal replacement products, Cambridge Weight Plan) for eight weeks [25]. Participants who achieved a weight loss of ≥ 5% were randomly assigned to one of four groups for one year: exercise and placebo (exercise); liraglutide and habitual activity (liraglutide); exercise and liraglutide (combination); or placebo and habitual activity (placebo).

The exercise intervention was designed to meet the WHO recommendations on physical activity: a minimum of 150 min/week of moderate-intensity or 75 min/week of vigorous-intensity aerobic physical activity or an equivalent combination to reach adequate exercise volume (duration × intensity). Participants randomized to exercise were encouraged to attend supervised group sessions twice a week and perform exercise individually twice a week. Exercise was targeted at 80% of maximal heart rate, and heart rate monitors were worn at all exercise sessions to assess adherence. Participants not randomized to exercise were instructed to maintain habitual physical activity until the end of the trial. Details on the exercise intervention have been reported elsewhere [24].

Study medication, liraglutide 6 mg/mL (Saxenda), or volume-matched placebo was injected subcutaneously via pens by the participants, commencing at 0.6 mg/day with weekly increments of 0.6 mg/day after consultation, eventually reaching 3.0 mg/day. Participants who had unacceptable adverse events at the targeted dose received the maximally tolerated dose at which they did not have such events. Participants remained enrolled if the medication was discontinued [25].

### Outcomes

MetS-Z (metabolic syndrome severity z-score) was a prespecified secondary endpoint in the trial protocol [24]. MetS-Z was developed by Gurka, DeBoer, and colleagues and the Clinical and Translational Science – Informatics and Technology group, University of Florida, and is based on the National Health and Nutrition Examination Survey (NHANES) from 1999 to 2010 (a representative sample of the US national household population) [11]. MetS-Z can be interpreted as a z-score normally distributed with a mean of 0 and a standard deviation of 1. Applied to the present study, the score shows how many standard deviations a given participant’s MetS score is from the NHANES population mean [11, 27]. MetS-Z was calculated in the participants with a value for all five factors at a given visit using the sub-group coefficients for non-Hispanic white men and women older than 20 years of age; see Additional file 1 for details [11].

The participants were also scored on the traditional MetS factors according to the harmonized metabolic syndrome definition: Waist circumference > 94 cm (in males) and > 80 cm (in females), HDL-c < 1.0 mmol/L in males and < 1.3 mmol/L in females, triglycerides ≥ 1.7 mmol/L, fasting glucose ≥ 5.6 mmol/L, and systolic blood pressure ≥ 130 mmHg or diastolic blood pressure ≥ 85 mmHg [8]. Participants were classified as having MetS if three or more factor cut-offs were exceeded. MetS-Z and MetS factors were only included for participants with a complete dataset (i.e., a value for all five factors at a given visit). The homeostatic model assessment of insulin resistance (HOMA-IR), an index of insulin resistance, was calculated by multiplying fasting insulin levels with fasting glucose levels, divided by 22.5 (see Additional file 1 for details on the calculation of HOMA-IR).

The procedure for blood samples, anthropometric measurements, and blood pressure is reported in the protocol article of the trial [25].

Dual-energy x-ray absorptiometry (Hologic, Discovery A) full-body scans were used to assess body composition in the fasting state. Fat mass, including android fat (an estimate of abdominal fat) and gynoid fat (an estimate of gluteofemoral fat), were determined by the scanner using APEX System Software Version 3.4.2; see Additional file 1 for details.

The inflammation marker hsCRP was assessed using V-PLEX Vascular Injury Panel 2 (human) Kits (MDS MULTI-SPOT Assay System). Only complete data sets were analyzed (i.e., participants with a blood sample from all three visits) for hsCRP.

Outcomes were obtained before the low-calorie diet (at week -8), after the low-calorie diet (week 0, at randomization), and at the end of the trial (week 52). Adverse events were registered at all visits and have previously been published [24].

### Statistical analysis

Continuous variables are summarized as means with ± standard deviations (± SD) or medians with interquartile range. Continuous outcomes with repeated measures were analyzed using a mixed linear model in the per-protocol population (i.e., the 130 participants adherent to the prescribed interventions), which might provide a better mechanistic understanding of the interventions, and in the intention-to-treat population (i.e., all 195 participants randomized). Significance testing was performed using α = 0.05 on MetS-Z, android fat percentage, and hsCRP outcomes. The following fixed effects were included in the model: time (factorial), group, age group (< / ≥ 40 years), sex, a time-group interaction, and a repeated effect for visit. A supplementary analysis further adjusting for blood pressure or lipid-lowering medication, smoking, and alcohol consumption at inclusion was also performed. All missing data were assumed to be missing at random. The analyses were unadjusted for multiplicity; therefore, definite inferences cannot be made. Results are reported as estimated changes with 95% confidence intervals (95% CI). Statistical sample size power analysis has previously been published and was based on body weight change (a 4 kg difference between the four groups was estimated to require at least 30 participants per group) [24]. All analyses were performed in SAS version 9.4 using SAS Enterprise Guide 7.1. Figures were made in R (3.6.2).

Regarding hsCRP, the non-normal distributed results were log-transformed before analysis and back-transformed as ratios with 95% CI. Three samples were excluded before analysis due to sample dilution error or hsCRP values consistent with concurrent infection or other diseases.

## Results

### Study population

At inclusion, before the low-calorie diet, the study population was 215 participants (63% women), 42 ± 12 years of age, and a mean BMI of 37.0 ± 2.9. See baseline characteristics in Table 1 and Additional file 1: Table S1. Smoking and alcohol consumption at inclusion are shown in Additional file 1: Table S2.

**Table 1:** Baseline characteristics

	Before low-calorie diet (n = 215)	After low-calorie diet (at randomization) (n = 195)	Estimated changes (n = 195)
Male/Female, n (%)	80/135 (37/63)	71/124 (36/64)	
Age, years	42 ± 12	43 ± 12	
Hypertension^a^, n (%)	134 (62)	63 (33)	
Pre-diabetes^b^, n (%)	96 (45)	30 (15)	
Blood pressure medication, n (%)	26 (12)		
Lipid-lowering medication, n (%)	14 (6)		
Metabolic syndrome			
Waist circumference^c^, cm	110.6 ± 11.3	100.3 ± 10.0	− 10.6 (− 11.4 to − 9.9)
Systolic blood pressure^c^, mmHg	132 ± 16	122 ± 13	− 10 (− 12 to − 8)
Diastolic blood pressure^c^, mmHg	86 ± 9	79 ± 8	− 7 (− 9 to − 6)
HDL− c^c^, mmol/L	1.3 ± 0.3	1.1 ± 0.3	− 0.1 (− 0.2 to − 0.1)
Triglycerides^c^, mmol/L	1.5 ± 1.0	1.1 ± 0.4	− 0.4 (− 0.5 to − 0.3)
Fasting glucose, mmol/L	5.6 ± 0.6	5.2 ± 0.5	− 0.5 (− 0.6 to − 0.4)
HOMA-IR^c,d^	3.9 ± 2.4	1.7 ± 1.0	0.44 (0.41 to 0.48)
MetS-Z, score	0.57 ± 0.59	0.06 ± 0.49	− 0.52 (− 0.58 to − 0.45)***
Body composition			
Body weight^c^, kg	109.7 ± 14.9	96.7 ± 12.5	− 13.1 (− 13.7 to − 12.4)
BMI^c^, kg/m^2^	37.0 ± 2.9	32.6 ± 2.9	− 4.4 (− 4.5 to − 4.2)
Total fat percentage^c^, %-points	41.0 ± 6.1	38.6 ± 6.9	− 2.4 (− 2.6 to − 2.1)
Android fat percentage, %-points Female, %-points Male, %-points	44.3 ± 4.7 46.0 ± 4.1 41.3 ± 4.2	41.4 ± 6.0 44.0 ± 4.8 36.8 ± 5.1	− 2.9 (− 3.4 to − 2.5)*** − 2.0 (− 2.5 to − 1.4)*** − 4.6 (− 5.2 to − 3.9)***
Gynoid fat percentage, %-points Female, %-points Male, %-points	40.7 ± 7.3 45.0 ± 4.3 32.9 ± 4.6	38.9 ± 7.7 43.6 ± 4.6 30.7 ± 4.5	− 1.8 (− 2.1 to − 1.5) − 1.5 (− 1.8 to − 1.1) − 2.3 (− 2.8 to − 1.9)
Android-gynoid ratio	1.11 ± 0.16	1.09 ± 0.15	− 0.03 (− 0.04 to − 0.02)
Inflammation marker			
hsCRP^e^mg/L	3.8 (1.6 to 8.4)	2.4 (1.0 to 5.6)	0.68 (0.59 to 0.78)***

Values are observed mean ± standard deviation. Changes are estimated mean differences (95% confidence intervals). Significance testing was only performed on MetS-Z, android fat percentage, and hsCRP. The results are adjusted for age group (< / ≥ 40 years) and sex

*MetS-Z* metabolic syndrome severity score, *BMI* Body Mass Index, *hsCRP* high-sensitivity C-reactive protein

^a^Hypertension is defined as systolic blood pressure > 130 mmHg or diastolic blood pressure > 85 mmHg

^b^Pre-diabetes is defined as fasting glucose > 5.6 mmol/L

^c^Outcomes previously reported [24]

^d^HOMA-IR: homeostatic model assessment of insulin resistance, calculated as fasting insulin times fasting glucose levels, divided by 22.5. Change is shown as the geometric mean ratio (95% confidence intervals)

^e^Median with interquartile range; change in hsCRP is presented as ratio (95% confidence intervals) via back-transformed log-data

***p < 0.001

**Fig. 1:**
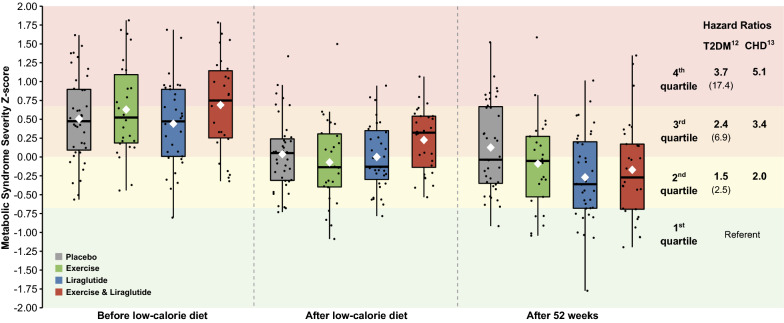
Observed MetS-Z before and after Low-calorie Diet and at Week 52 by Randomization Group. Observed MetS-Z of individual per-protocol participants (black dots) by randomization group at the three visits, before the low-calorie diet (week -8), after the low-calorie diet (week 0), and end of the trial (week 52), presented as box plots. Tops of the boxes indicate the upper quartile; bottom of the box is the lower quartile; white diamonds observed mean; black horizontal line medians; whiskers ± 1.5 times the interquartile range or highest or smallest observation. Box plots overlay MetS-Z quartiles associated with the risk of future diabetes [12] and coronary heart disease [13] compared to the first quartile and adjusted for individual MetS factors. For diabetes, unadjusted risks are also shown in parentheses. *MetS-Z* metabolic syndrome severity z-score. *T2DM* type 2 diabetes, *CHD* coronary heart disease

The observed mean MetS-Z was at inclusion 0.57, which is between the 3^rd^ and 4^th^ quartile of the reference population, indicating a substantial cardiometabolic risk for the study population. MetS-Z quartiles and their associated risks are presented in Fig. 1 of this study. At inclusion, the mean MetS-Z of female participants was placed within the 3^rd^ quartile of MetS-Z scores, while the mean for males was on the border of the 3^rd^ and 4^th^ quartiles. The distributions of scores between the groups were similar. The pattern of change in MetS-Z was generally similar between men and women between the three visits (see Additional file 1: Figs. S2 and S3 for observed MetS-Z for women and men separately). At inclusion, 62% of participants had hypertension, and 45% had pre-diabetes.

A total of 166 participants (85%) completed the study by attending final assessments at week 52. Thus, 15% were lost to follow-up (placebo: 9, exercise: 8, liraglutide: 8, combination: 4), Additional file 1: Fig. S1. Overall, there was an even pattern of loss to follow-up, and the most common cause of dropout was personal life conditions (e.g., job-related changes). The per-protocol population included 130 participants (placebo = 39; exercise = 26, liraglutide = 36; combination = 29).

Changes in body weight and total body fat percentage have previously been published [24]. In summary, results from the trial show that after the low-calorie diet, the participants had reduced body weight by 13.1 kg (~ 12%), Table 1. After one year, the placebo group had increased body weight. The exercise and liraglutide groups maintained body weight while lowering the total fat percentage. The combination group decreased body weight and fat percentage (Table 2) [24].

**Table 2:** Changes from randomization to week 52

	Placebo	Exercise	Liraglutide	Combination
	(n = 39)	(n = 26)	(n = 36)	(n = 29)
Metabolic syndrome				
Waist circumference, cm	4.6 (2.4 to 6.7)	− 0.3 (− 3.0 to 2.4)	− 1.2 (− 3.5 to 1.0)	− 6.3 (− 8.8 to − 3.8)
Systolic blood pressure, mmHg	4.3 (− 0.4 to 9.1)	3.6 (− 2.2 to 9.5)	− 0.7 (− 5.7 to 4.3)	1.2 (− 4.3 to 6.8)
Diastolic blood pressure, mmHg	3.0 (0.3 to 5.6)	1.1 (− 2.2 to 4.3)	− 0.4 (− 3.1 to 2.4)	− 0.1 (− 3.2 to 3.0)
HDL- c, mmol/L	0.25 (0.18 to 0.32)	0.24 (0.16 to 0.32)	0.26 (0.19 to 0.33)	0.31 (0.24 to 0.39)
Triglycerides, mmol/L	0.0 (− 0.1 to 0.2)	0.2 (0.0 to 0.3)	0.0 (− 0.1 to 0.2)	0.1 (0.0 to 0.3)
Fasting glucose, mmol/L	0.4 (0.2 to 0.6)	0.1 (− 0.1 to 0.4)	− 0.2 (− 0.4 to 0.0)	− 0.2 (− 0.4 to 0.0)
HOMA-IR^a^	1.55 (1.32 to 1.82)	1.19 (0.98 to 1.45)	1.34 (1.13 to 1.59)	1.02 (0.85 to 1.23)
MetS-Z, score	0.09 (− 0.06 to 0.23)	− 0.03 (− 0.21 to 0.16)	− 0.28 (− 0.43 to − 0.13)*** ^§^	− 0.39 (− 0.56 to − 0.22)***^§^
Participants with Hypertension^b^, n (%)	19 (49)	12 (46)	13 (36)	7 (24)
Pre diabetes^c^, n (%)	14 (36)	6 (23)	3 (8)	4 (14)
Body composition				
Body weight^d^, kg	6.1 (3.4 to 8.7)	0.7 (− 2.5 to 3.9)	− 1.9 (− 4.6 to 0.8)	− 6.0 (− 9.0 to − 3.0)
Total fat percentage^d^, %-points	0.3 (− 1.0 to 1.7)	− 1.8 (− 3.2 to − 0.4)	− 1.9 (− 3.1 to − 0.7)	− 3.7 (− 4.9 to − 2.4)
Android fat percentage, %-points	0.1 (− 1.3 to 1.4)	− 2.5 (− 4.2 to − 0.8)**^§^	− 2.8 (− 4.2 to − 1.3)**^§^	− 6.1 (− 7.7 to − 4.4)***^§^
Female, %-points	− 0.4 (− 2.2 to 1.4)	− 3.6 (− 5.9 to − 1.4)**^§^	− 3.3 (− 5.1 to − 1.5)***^§^	− 6.4 (− 8.5 to − 4.4)***^§^
Male, %-points	0.8 (− 1.5 to 30)	− 1.0 (− 3.6 to 1.6)	− 1.9 (− 4.3 to 0.5)	− 5.4 (− 8.0 to − 2.8)***^§^
Gynoid fat percentage, %- points	0.3 (− 0.6 to 1.2)	− 1.7 (− 2.7 to − 0.6)	− 1.1 (− 2.1 to − 0.2)	− 3.8 (− 4.8 to − 2.8)
Female, %-points	0.0 (− 1.1 to 1.2)	− 1.9 (− 3.4 to − 0.4)	− 1.2 (− 2.4 to 0.0)	− 3.8 (− 5.1 to − 2.4)
Male, %-points	0.7 (− 0.7 to 2.1)	− 1.3 (− 2.9 to 0.4)	− 1.1 (− 2.6 to 0.5)	− 3.8 (− 5.4 to − 2.1)
Android-gynoid ratio	− 0.01 (− 0.03 to 0.02)	− 0.02 (− 0.04 to 0.01)	− 0.05 (− 0.07 to − 0.02)	− 0.06 (− 0.08 to − 0.03)
Inflammation marker				
hsCRP^e^	0.85 (0.61 to 1.20)	0.82 (0.55 to 1.22)	0.64 (0.45 to 0.92)*	0.48 (0.33 to 0.71)***^§^

Per-protocol analysis. Changes are estimated mean differences (95% confidence intervals) within-group. Significance testing was only performed on MetS-Z, android fat percentage, and hsCRP. The results are adjusted for age group (< / ≥ 40 years) and sex

*MetS-Z* metabolic syndrome severity score. *hsCRP* high-sensitivity C-reactive protein

^a^HOMA-IR: homeostatic model assessment of insulin resistance, calculated as fasting insulin times fasting glucose levels, divided by 22.5. Change is shown as geometric mean ratios (95% confidence intervals)

^b^Hypertension: systolic blood pressure > 130 mmHg or diastolic blood pressure > 85 mmHg

^c^Pre-diabetes: fasting glucose > 5.6 mmol/L

^d^Outcomes previously reported [24]

^e^Changes are presented as ratios (95% confidence intervals) via back-transformed log-data

*p < 0.05, **p < 0.01, and ***p < 0.001 within-group

**Fig. 2:**
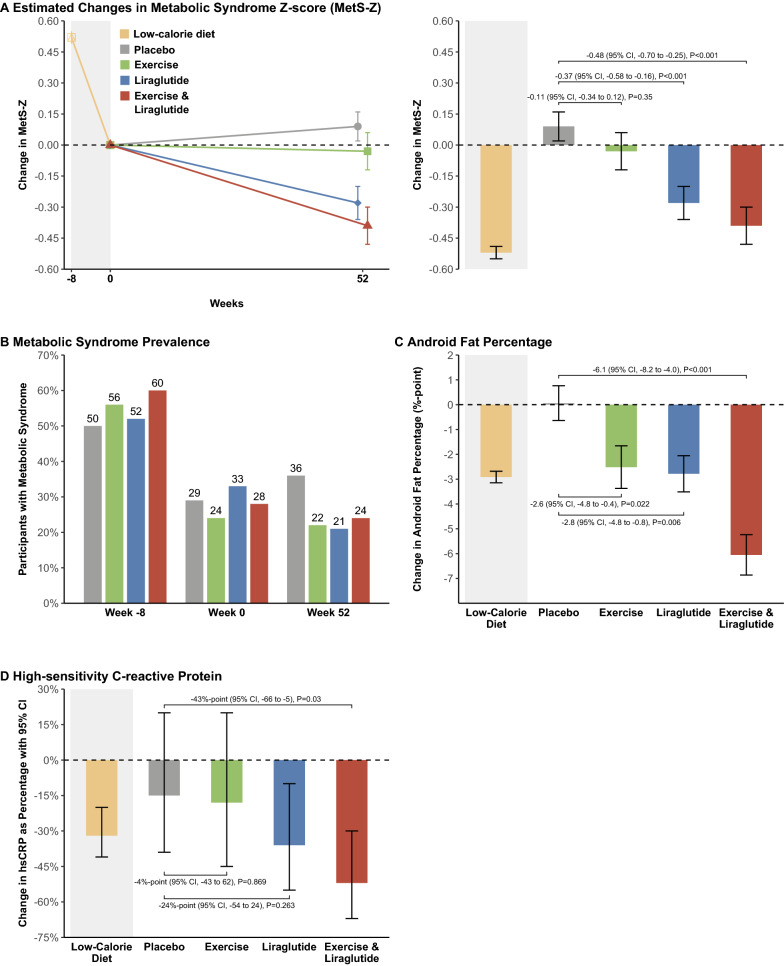
Changes During Low-calorie Diet and From Randomization to Week 52. Per-protocol analysis of mixed model estimated changes in metabolic syndrome severity z-score (**A**), metabolic syndrome prevalence (**B**), android fat percentage (**C**), and high-sensitivity C-reactive protein (**D**) during a low-calorie diet (shaded area; weeks -8 to 0) and treatment (weeks 0 to 52). Changes are estimated mean differences with ± standard error of the mean. Changes in high-sensitivity C-reactive protein are presented as percentages via ratios from back-transformed log-data and shown with 95% confidence intervals. Between-group changes are estimated mean differences with 95% confidence intervals and p-values. Results are adjusted for age group (< / ≥ 40 years) and sex. Dashed line is the baseline for the low-calorie diet and randomized groups (at week 0)

^§^p < 0.05 vs. placebo (see Fig. 2 for details on between-group changes)

In the following Results section, we present the results from the participants who completed the trial according to the prescribed interventions (Table 2 and Additional file 1: Table S3 and Fig. 2). The intention-to-treat analysis, including all randomized participants, is presented in Additional file 1: Table S4.

### Changes in metabolic syndrome

The MetS-Z decreased by 0.52 to 0.06, P < 0.001, during the low-calorie diet (Table 1 and Fig. 2A). This reduction shifted the Mets-Z means of all groups from the top 3^rd^ and bottom 4^th^ quartiles of the reference population, which indicates higher risk of diabetes and coronary heart disease, to average close to the limit of the 2^nd^ and 3^rd^ quartiles, which indicates a lower risk of diabetes and coronary heart disease after the low-calorie diet (Fig. 1). The diet-induced mean changes of individual MetS factors are shown in Table 1, which collectively translated into a decreased average prevalence of MetS from 55 to 29% after the low-calorie diet (Fig. 2B). Furthermore, the prevalence of participants with hypertension was halved (from 62 to 33%), while the prevalence of pre-diabetes was reduced by two-thirds (from 45 to 15%). Insulin resistance, measured by HOMA-IR, was 3.9 ± 2.4 before the low-calorie diet was reduced by 56% to 1.7 ± 1.0 after the diet (Table 1).

One year after the low-calorie diet, the MetS-Z was unchanged in the placebo and exercise groups (Table 2). Compared to placebo, MetS-Z decreased by 0.37, P < 0.001, in the liraglutide group and by 0.48, P < 0.001, in the combination group (Fig. 2A). Noticeably, the means of MetS-Z in the liraglutide and combination groups moved from the higher risk 3^rd^ to the lower risk 2^nd^ quartile, indicating a further risk reduction on top of the risk reduction by the low-calorie diet (Fig. 1). The prevalence of participants with MetS at week 52 was similar across active treatment groups, whereas the prevalence was higher within the placebo group (Fig. 2B). The prevalence of participants with hypertension or pre-diabetes was generally lower in the active treatment groups and notably lowered in the groups treated with liraglutide.

Adjusting for blood pressure or lipid-lowering medication, smoking, and alcohol consumption at inclusion did not affect the analysis results (Additional file 1: Table S5).

The reduced insulin resistance seen during low-calorie was maintained in the adherent exercise groups, while insulin resistance increased in the placebo and liraglutide groups (Table 2) after one year.

### Changes in fat distribution

Android fat percentage was 44.3% ± 4.7 before the low-calorie diet and decreased by 2.9%-points, P < 0.001 to 41.4% ± 6.0 after the diet (Table 1). See Additional file 1: Table S6 for absolute masses. Men had a lower android fat percentage than women (41.3 vs. 46.0%, respectively) at inclusion and had larger reductions of android fat percentage than women during the low-calorie diet (− 4.6 vs. − 2.0%-points, respectively).

After one year, android and gynoid fat percentages were unchanged in the placebo group; however, android and gynoid fat masses increased (Table 2 and Additional file 1: Table S7). Generally, the active treatment groups seemed to lose relatively more android fat than gynoid fat. Compared to the placebo group, the exercise group decreased android fat percentage by 2.6%-points, P = 0.022, and the liraglutide group decreased android fat percentage by 2.8%-points, P = 0.006, Fig. 2C. Thus, participants in the exercise and liraglutide groups decreased android fat percentage by around 6%-points during the entire trial. The combination group decreased android fat percentage by 6.1%-points, P < 0.001, compared to placebo, around twice as much as exercise or liraglutide treatment alone. Furthermore, in men, android fat percentage was only significantly decreased in the combination group, whereas in women, android percentage was reduced in all the active treatment groups (Table 2).

### Changes in high-sensitivity C-reactive protein

The median concentration of the inflammation marker hsCRP was 3.8 mg/L before the low-calorie diet and decreased by 32% to 2.4 mg/L after the diet, P < 0.001, Table 1.

After one year, the hsCRP concentrations did not change in the placebo and exercise groups (Table 2 and Fig. 2D). Within the liraglutide group, hsCRP decreased by 36%; however, this decrease was not different from the placebo group. The combination group reduced hsCRP by 43% compared to the placebo group, P = 0.030. In the intention-to-treat analysis, hsCRP decreased by 35% within the combination group, but this change was not different from the placebo group (Additional file 1: Table S4).

### Adherence to interventions

In the per-protocol population, the exercise group performed 156 ± 54 min/week at an intensity of 78 ± 4% of maximum heart rate, and the combination group performed 144 ± 67 min/week at 78 ± 5% of maximum heart rate. The average dose of study medication was at least 2.6 mg/day in all groups. Details regarding exercise and study medication adherence in the intention-to-treat population have previously been published [24].

### Safety

Gastrointestinal adverse events (e.g., one or more experiences of nausea, diarrhea, or vomiting during one year) were more commonly reported in the groups receiving liraglutide (placebo group: 45%, exercise group: 65%, liraglutide group: 86%, combination group: 71%). The frequency of serious adverse events was 4%, 8%, 12%, and 8% in the placebo, exercise, liraglutide, and combination groups, respectively. All safety outcomes have previously been reported [24].

## Discussion

Identifying and managing the risk of cardiometabolic disease associated with obesity remains a major healthcare challenge. Metabolic syndrome, abdominal obesity, and low-grade inflammation constitute risk factors for future cardiometabolic disease. Therefore, we investigated improvements in metabolic syndrome, abdominal obesity, and low-grade inflammation during exercise, a glucagon-like peptide 1 receptor agonist, or the combination of the two following an eight-week low-calorie diet.

The diet-induced weight loss reduced MetS-Z, abdominal obesity, and inflammation marker hsCRP. After one year, the combination of exercise and liraglutide treatment reduced MetS-Z, android fat percentage, and hsCRP compared to placebo. Exercise treatment maintained MetS-Z and hsCRP and reduced android fat percentage compared to placebo. Liraglutide treatment reduced Mets-Z and android fat percentage while maintaining hsCRP compared to placebo. Placebo treatment was associated with maintenance of the diet-induced reductions in MetS-Z, hsCRP, and android fat percentage, even though 50% of the weight lost during the low-calorie diet was regained in the placebo group, while MetS prevalence and fat masses increased again. In addition, we have previously reported that the placebo group became sedentary one year after the initial weight loss [28].

Large reductions in MetS-Z, abdominal obesity, and hsCRP compared to placebo were seen in the combination group, providing large potential reductions in cardiometabolic risk. Furthermore, the combination group showed a reduction of android fat percentage that was about twice as large as the reduction seen in both the exercise and liraglutide groups, underlining the complementary effects of combined treatment.

Liraglutide treatment alone further reduced MetS-Z following the diet-induced reductions, largely due to reduced fasting glucose, an expected effect of liraglutide treatment [29]. Similar decreases in android fat percentage were seen with exercise compared to the liraglutide group, but exercise did not further reduce MetS. Exercise has been found to decrease MetS, but these studies did not include an initial diet-induced weight loss phase [16, 30]. These findings suggest that, in already weight-reduced individuals, treatment with GLP-1 RA might be helpful in reducing cardiometabolic risk further.

If the comparative risks, illustrated in Fig. 1, indeed reflect the risks of the participants, the liraglutide groups substantially reduced the risk of future diabetes and coronary heart disease. Clinically, the MetS-Z model might prove valuable in guiding the primary prevention of cardiometabolic disease.

The combination group reduced body weight due to fat mass loss with a preferential reduction of android fat rather than gynoid fat. Thus, during the entire trial, abdominal obesity of participants in the combination group was reduced by almost 8%-points while maintaining total lean mass. This finding contrasts with other weight loss strategies, including the low-calorie diet used in this trial, which often lead to large amounts of lost lean mass (e.g., 20–50% lost by bariatric surgery, 30–47% by GLP-1 RA treatment before weight loss) [31]. Furthermore, in men, only the combination treatment was able to lower android fat percentage.

The liraglutide group reduced android fat percentage without changing body weight, suggesting a reduction of android fat percentage independent of weight loss. This is consistent with recent findings from a 36-week study examining changes to visceral fat estimated by magnetic resonance imaging in response to treatment with liraglutide [32]. Regarding hsCRP, we observed a significant reduction in the combination compared to the placebo group in the per-protocol analysis. In the intention-to-treat analysis, this reduction was no longer significantly different from the placebo group. This suggests that adherent exercise in combination with liraglutide might be able to add improvements to low-grade inflammation.

Despite similar reductions in android fat in the exercise and liraglutide group, exercise did not significantly decrease hsCRP after one year. However, studies that demonstrate reduced inflammation as an effect of exercise do often not have an initial weight loss phase [17, 33], which substantially reduced hsCRP in this study (from a median of 3.8 to 2.4 mg/L); thus, the possibility for additional improvement through physical activity alone might have been limited.

In a clinical setting, hsCRP levels higher than 3 mg/L indicate increased cardiovascular risk [14, 15]. At inclusion in this study, the mean hsCRP level was above the upper limit of hsCRP and approached the lower limit of 1 mg/L in the liraglutide treatment groups at the end of the trial. When liraglutide treatment was combined with adherent exercise, hsCRP was reduced by more than 50% during the entire trial. Therefore, these hsCRP findings indicate that combination treatment can exert clinically meaningful reductions in low-grade inflammation after diet-induced weight loss. Regarding insulin resistance, adherent exercise was able to maintain the large reductions in HOMA-IR induced by the low-calorie diet.

A strength of this study is the longitudinal, randomized, placebo-controlled design with four separate groups to assess single and combined effects of treatments with exercise and liraglutide 3.0 mg/day. Another strength is the novelty of analyzing the effects of maintained interventions on a clinically relevant continuous metabolic syndrome score combined with assessments of abdominal obesity and inflammation, translating to potential cardiometabolic risk.

In this study, we present the findings from the participants who completed the trial according to the prescribed interventions to better observe the effects of actually performed exercise, often confounded by inadequate adherence [19]. The limitation of this approach includes a possible selection bias which might have skewed the treatment estimate. A reason for not fulfilling the high demands of the per-protocol requirements may be the time consumed on exercise, which is a known barrier to exercise [34]. In the present study, the per-protocol participants in the exercise groups performed an average of 2.5 h of exercise per week for a whole year. Importantly, we also present the intention-to-treat analysis, including the 36 participants not fulfilling the high per-protocol demands, which generally painted a similar picture in the placebo, exercise, and liraglutide groups compared to the per-protocol analysis. Thus, except for hsCRP, which only showed significant differences between the placebo and combination groups in the per-protocol population, there were no differences in the results between intention-to-treat or per-protocol analyses.

## Conclusion

In people with obesity at risk of developing cardiometabolic disease, the low-calorie diet improved MetS-Z, abdominal obesity, and inflammation marker hsCRP. After one year, intervention with exercise further reduced abdominal obesity, liraglutide treatment further reduced MetS-Z and abdominal obesity, and liraglutide combined with adherent exercise further reduced MetS-Z, abdominal obesity as well as hsCRP compared to placebo. The combination treatment thereby reduced all outcomes compared to placebo, potentially providing the largest risk reductions of future cardiometabolic disease in an adult population with obesity.

## Supplementary Information

**Additional file 1: Table S1.** Characteristics of Completers at Randomization. **Table S2.** Medication, Smoking and Alcohol Consumption before the Low-calorie Diet by Randomization Group. **Table S3.** Estimated Treatment Differences vs. Placebo Group at Week 52 – Per-protocol Population. **Table S4.** Changes from Randomization to Week 52 – Intention-to-treat Population. **Table S5.** Supplementary Analysis of Changes in MetS-Z from Randomization to Week 52. **Table S6.** Absolute Changes from Week -8 to 0. **Table S7.** Changes in Absolute Masses from Randomization to Week 52 – Per-protocol Population. **Figure S1.** CONSORT flow diagram. **Figure S2.** Observed MetS-Z of Women by Randomization Group. **Figure S3.** Observed MetS-Z of Men by Randomization Group.

## Data Availability

The study protocol and statistical analysis plan have been published [24, 25]. De-identified data under the General Data Protection Regulations (GDPR) may be available for research collaboration purposes upon reasonable request to the corresponding author (Signe Sørensen Torekov, torekov@sund.ku.dk) and will require the completion of a data processing agreement.

## Abbreviations

MetS: Metabolic syndrome
MetS-Z: Metabolic syndrome severity z-score
hsCRP: High-sensitivity c-reactive protein
GLP-1 RA: Glucagon-like peptide 1 receptor agonist
HR: Hazard ratio
