# Supplementary material for: Combination of exercise and GLP-1 receptor agonist treatment reduces severity of metabolic syndrome, abdominal obesity, and inflammation: a randomized controlled trial
Source: Cardiovasc Diabetol. 2023 Feb 25;22:41. doi: 10.1186/s12933-023-01765-z (PMC9960425; doi:10.1186/s12933-023-01765-z)
Supplement: Supplementary file 1 — Additional file 1: Table S1. Characteristics of Completers at Randomization. Table S2. Medication, Smoking and Alcohol Consumption before the Low-calorie Diet by Randomization Group. Table S3. Estimated Treatment Differences vs. Placebo Group at Week 52 – Per-protocol Population. Table S4. Changes from Randomization to Week 52 – Intention-to-treat Population. Table S5. Supplementary Analysis of Changes in MetS-Z from Randomization to Week 52. Table S6. Absolute Changes from Week -8 to 0. Table S7. Changes in Absolute Masses from Randomization to Week 52 – Per-protocol Population. Figure S1. CONSORT flow diagram. Figure S2. Observed MetS-Z of Women by Randomization Group. Figure S3. Observed MetS-Z of Men by Randomization Group. [file 12933_2023_1765_MOESM1_ESM.docx]

## Additional file

## Results

| **Table S1: Characteristics of Completers at Randomization** | **All groups** | **Placebo** | **Exercise** | **Liraglutide** | **Combination** |
| --- | --- | --- | --- | --- | --- |
| **Per-protocol participants (n)** | 130 | 39 | 26 | 36 | 29 |
| **Age**, years | 44.8±11.7 | 43.7±12.3 | 45.0±11.8 | 46.2±10.3 | 44.5±12.7 |
| **Sex,** n (men/women) | 50/80 | 15/24 | 11/15 | 13/23 | 11/18 |
| **BMI**, kg/m^2^ | 32.4±2.9 | 32.1±2.9 | 32.2±3.3 | 32.5±3.1 | 32.6±2.5 |
| **Body weight**, kg | 96.5±12.3 | 96.6±13.2 | 96.1±13.2 | 94.8±11.3 | 99.1±11.4 |
| **MetS-Z,** score | 0.05±0.49 | 0.04±0.48 | -0.07±0.58 | 0.00±0.44 | 0.23±0.42 |
| **Android fat percentage**, %-points | 40.7±6.0 | 40.6±6.1 | 38.2±6.2 | 41.6±5.5 | 41.9±5.8 |
| **hsCRP**^§^ (mg/L) | 2.4 (1.0 to 5.5) | 2.4 (1.0 to 5.3) | 2.4 (1.4 to 5.3) | 1.8 (0.9 to 4.1) | 3.6 (1.3 to 6.5) |
|  |  |  |  |  |  |
| **Non-per-protocol participants (n)** | 36 | 1 | 14 | 5 | 16 |
| **Age**, years | 41.3±11.4 | 39.5 | 44.7±11.3 | 37.7±14.3 | 39.6±10.9 |
| **Sex,** n (men/women) | 11/25 | 0/1 | 4/10 | 1/4 | 6/10 |
| **BMI**, kg/m^2^ | 32.8±2.4 | 37.9 | 32.6±2.3 | 31.8±2.0 | 32.9±2.5 |
| **Body weight**, kg | 96.4±13.7 | 105.0 | 97.6±13.0 | 90.5±21.8 | 96.7±12.1 |
| **MetS-Z,** score | 0.01±0.44 | 0.12 | 0.09 ±0.25 | 0.15±0.55 | -0.12±0.53 |
| **Android fat percentage**, %-points | 43.0±5.5 | 38.0 | 43.0±4.4 | 43.9±4.5 | 42.9±6.8 |
| **hsCRP**^§^ (mg/L) | 2.4 (1.0 to 5.7) | NA | 3.2 (2.1 to 6.8) | 1.6 (1.2 to 5.3) | 1.2 (0.6 to 4.4) |
| Values are observed mean±standard deviation at randomization (week 0) of participants who completed the trial per-protocol and participants who completed the trial but not per-protocol. ^§^Median with interquartile range. NA: hsCRP data was missing for the one participant in the placebo group of non-adherent completers. MetS-Z: metabolic syndrome severity score. hsCRP: high-sensitivity C-reactive protein. | | | | | |

| **Table S2: Medication, Smoking and Alcohol Consumption before the Low-calorie Diet by Randomization Group** | | | | |
| --- | --- | --- | --- | --- |
|  | **Placebo** | **Exercise** | **Liraglutide** | **Combination** |
| **Blood pressure medication,** n (%) | 7 (14) | 5 (10) | 3 (6) | 9 (18) |
| **Lipid-lowering medication,** n (%) | 3 (6) | 4 (8) | 1 (2) | 6 (12) |
| **Currently smoking,** n (%) | 7/44 (16) | 7/40 (18) | 6/41 (15) | 4/42 (10) |
| **Previously smoked,** n (%) | 15/35 (43) | 15/33 (45) | 14/34 (41) | 17/37 (46) |
| **Alcohol consumption**, average standard drinks/week ±SD | 3.5±4.8 | 2.8±3.8 | 2.2±5.2 | 3.4±6.1 |
| Blood pressure or lipid-lowering medication, smoking and alcohol consumption (average units per week during the last 12 months) before the low-calorie diet by randomization group. One participant in the exercise group consumed 70 standard drinks/week and was not included in the alcohol consumption mean in this table. SD: standard deviation. | | | | |

| **Table S3: Estimated Treatment Differences vs. Placebo Group at Week 52 – Per-protocol Population** | | | |
| --- | --- | --- | --- |
|  | **Exercise group vs Placebo group** | **Liraglutide group vs Placebo Group** | **Combination group vs Placebo group** |
| **MetS-Z**, score | -0.11 (-0.34 to 0.12), 0.349 | -0.37 (-0.58 to -0.16), <0.001 | -0.48 (-0.7 to -0.25), <0.001 |
| **Android fat percentage**, %-points | -2.6 (-4.8 to -0.4), 0.022 | -2.8 (-4.8 to -0.8), 0.006 | -6.1 (-8.2 to -4.0), <0.001 |
| **hsCRP**^a^, mg/L | 0.96 (0.57 to 1.62), 0.869 | 0.76 (0.46 to 1.24), 0.263 | 0.57 (0.34 to 0.95), 0.030 |
| Per-protocol analysis. The results are adjusted for age group (</≥ 40 years) and sex. Changes are estimated mean difference (95% confidence intervals), p-value. ^a^Changes are presented as ratios (95% confidence intervals), p-value, via back-transformed log-data. MetS-Z: metabolic syndrome severity z-score. hsCRP: high-sensitivity C-reactive protein. | | | |

| **Table S4: Changes from Randomization to Week 52 – Intention-to-treat Population** | | | | |
| --- | --- | --- | --- | --- |
|  | **Placebo** | **Exercise** | **Liraglutide** | **Combination** |
|  | (n = 49) | (n = 48) | (n = 49) | (n = 49) |
| **Metabolic syndrome** | | | | |
| **Waist circumference**†, cm | 4.4 (2.2 to 6.7) | 0.5 (-1.8 to 2.8) | -1.1 (-3.2 to 1.1) | -3.9 (-6.0 to -1.8) |
| **Systolic Blood pressure**†**,** mmHg | 4.2 (-0.3 to 8.6) | 3.6 (-0.9 to 8.1) | -1.0 (-5.4 to 3.4) | -0.1 (-4.4 to 4.2) |
| **Diastolic blood pressure**†**,** mmHg | 2.8 (0.2 to 5.4) | 1.1 (-1.5 to 3.7) | 0.1 (-2.5 to 2.7) | -0.3 (-2.8 to 2.2) |
| **HDL-c**†**,** mmol/L | 0.2 (0.1 to 0.3) | 0.3 (0.2 to 0.3) | 0.2 (0.2 to 0.3) | 0.3 (0.2 to 0.3) |
| **Triglycerides**†**,** mmol/L | 0.0 (-0.1 to 0.1) | 0.1 (0.0 to 0.3) | 0.0 (-0.1 to 0.1) | 0.1 (0.0 to 0.2) |
| **Fasting glucose,** mmol/L | 0.4 (0.2 to 0.5) | 0.2 (0.0 to 0.3) | -0.2 (-0.4 to -0.1) | -0.1 (-0.2 to 0.1) |
| **HOMA-IR**†^a^ | 1.55 (1.31 to 1.84) | 1.32 (1.10 to 1.57) | 1.33 (1.13 to 1.57) | 1.16 (0.99 to 1.37) |
| **MetS-Z,** score | 0.09 (-0.07 to 0.24) | -0.04 (-0.21 to 0.12) | -0.29 (-0.44 to -0.14)*** ^§^ | -0.24 (-0.38 to -0.09)** ^§^ |
| **Body composition** | | | | |
| **Body weight**†, kg | 6.1 (3.5 to 8.7) | 2.0 (-0.7 to 4.6) | -0.7 (-3.2 to 1.8) | -3.4 (-5.9 to -0.9) |
| **Total fat percentage**†, %-points | 0.4 (-0.6 to 1.5) | -1.7 (-2.8 to -0.6) | -1.6 (-2.6 to -0.6) | -3.5 (-4.5 to -2.5) |
| **Android fat percentage**, %-points  **Female**, %-points  **Male**, %-points | 1. (-1.4 to 1.5)   -0.4 (-2.2 to 1.4)  0.8 (-1.8 to 3.4) | -3.0 (-4.5 to -1.4)*** ^§^  -3.7 (-5.6 to -1.7)*** ^§^  -1.8 (-4.4 to 0.8) | -2.9 (-4.3 to -1.5)*** ^§^  -3.5 (-5.2 to -1.8)*** ^§^  -1.7 (-4.4 to 1.0) | -5.2 (-6.6 to -3.8)*** ^§^  -5.7 (-7.4 to -4.0)*** ^§^  -4.5 (-6.9 to -2)*** ^§^ |
| **Gynoid fat percentage**, %-points  **Female**, %-points  **Male**, %-points | 0.3 (-0.7 to 1.2)  0.1 (-1.1 to 1.3)  0.6 (-1.1 to 2.3) | -1.8 (-2.8 to -0.8)  -1.8 (-3.1 to -0.5)  -1.7 (-3.4 to 0.0) | -1.2 (-2.2 to -0.3)  -1.3 (-2.5 to -0.2)  -1.1 (-2.8 to 0.7) | -3.3 (-4.2 to -2.4)  -3.2 (-4.3 to -2.1)  -3.5 (-5.1 to -1.9) |
| **Android-gynoid ratio** | 0.0 (-0.03 to 0.02) | -0.03 (-0.05 to -0.01) | -0.05 (-0.07 to -0.02) | -0.05 (-0.07 to -0.03) |
| **Inflammation marker** | | | | |
| **hsCRP**^‡^ (mg/L) | 0.85 (0.59 to 1.2) | 0.76 (0.53 to 1.1) | 0.64 (0.45 to 0.90)* | 0.65 (0.47 to 0.90)* |
| Intention-to-treat analysis. The results are adjusted for age group (</≥ 40 years) and sex. Changes are estimated mean difference (95% confidence intervals) within group. Significance testing was only performed on MetS-Z, android fat percentage, and hsCRP. †Outcomes previously reported (1). ‡Changes are presented as ratios (95% confidence intervals) via back-transformed log-data. ^a^HOMA-IR: homeostatic model assessment of insulin resistance, calculated as fasting insulin times fasting glucose levels, divided by 22.5. Change as geometric mean ratios with 95% confidence intervals. MetS-Z: metabolic syndrome severity score. hsCRP: high-sensitivity C-reactive protein. *p<0.05, **p<0.01, and ***p<0.001 within-group. ^§^p<0.05 vs. placebo. | | | | |

| **Table S5: Supplementary Analysis of Changes in MetS-Z from Randomization to Week 52** | | | | |
| --- | --- | --- | --- | --- |
|  | **Placebo** | **Exercise** | **Liraglutide** | **Combination** |
|  | (n = 39) | (n = 26) | (n = 36) | (n = 29) |
| **Per-protocol Population** | | | | |
| **MetS-Z,** score | 0.10 (-0.07 to 0.26) | -0.05 (-0.26 to 0.15) | -0.28 (-0.46 to -0.11)** ^§^ | -0.48 (-0.67 to -0.29)*** ^§^ |
|  |  |  |  |  |
| **Intention-to-treat Population** | | | | |
| **MetS-Z,** score | 0.10 (-0.07 to 0.27) | -0.06 (-0.24 to 0.13) | -0.29 (-0.46 to -0.12)** ^§^ | -0.31 (-0.48 to -0.14)*** ^§^ |
| Supplementary analysis adjusted for age group (</≥ 40 years), sex, blood pressure and lipid-lowering medication, smoking, and alcohol consumption (average standard drinks per week during the last 12 months) at inclusion. Changes are estimated mean difference (95% confidence intervals) within-group. MetS-Z: metabolic syndrome severity score. *p<0.05, **p<0.01, and ***p<0.001 within-group. ^§^p<0.05 vs. placebo. | | | | |

| **Table S6:**  **Absolute Changes from Week -8 to 0** | **Before low-calorie diet**  (n = 215) | **After low-calorie diet**  (n = 195) | **Changes**  (n = 195) |
| --- | --- | --- | --- |
| **Lean mass^a^**, kg | 65.3±12.9 | 60.4±11.6 | -5.1 (-5.6 to -4.6) |
| **Fat mass^a^**, kg | 44.9±7.2 | 37.7±7.2 | -7.3 (-7.7 to -6.9) |
| **Android fat mass**, kg | 4.2±9.4 | 3.3±8.1 | -0.9 (-0.9 to -0.8) |
| **Gynoid fat mass**, kg | 7.3±1.7 | 6.2±1.6 | -1.1 (-1.2 to -1.1) |
| **Android lean mass**, kg | 5.2±1.1 | 4.7±0.9 | -0.6 (-0.6 to -0.5) |
| **Gynoid lean mass**, kg | 10.6±2.0 | 9.7±1.7 | -1.0 (-1.1 to -0.9) |
| Values are observed mean±standard deviation. The results are adjusted for age group (</≥ 40 years) and sex. Changes are estimated mean difference (95% confidence intervals). **^a^**Outcomes previously reported (1). | | | |

| **Table S7: Changes in Absolute Masses from Randomization to Week 52 – Per-protocol Population** | | | | |
| --- | --- | --- | --- | --- |
|  | **Placebo** | **Exercise** | **Liraglutide** | **Combination** |
|  | (n = 39) | (n = 26) | (n = 36) | (n = 29) |
| **Lean mass^a^**, kg | 2.9 (1.8 to 4.0) | 2.1 (1.0 to 3.3) | 0.0 (-1.0 to 1.1) | 0.5 (-0.6 to 1.5) |
| **Fat mass^a^**, kg | 2.6 (0.7 to 4.5) | -1.2 (-3.2 to 0.8) | -2.0 (-3.9 to -0.2) | -4.7 (-6.5 to -2.9) |
| **Android lean mass**, kg | 0.41 (0.27 to 0.54) | 0.22 (0.06 to 0.38) | 0.09 (-0.04 to 0.23) | 0.05 (-0.1 to 0.2) |
| **Gynoid lean mass**, kg | 0.69 (0.48 to 0.91) | 0.46 (0.19 to 0.72) | -0.07 (-0.3 to 0.15) | -0.02 (-0.27 to 0.23) |
| **Android fat mass**, kg | 0.29 (0.09 to 0.49) | -0.15 (-0.39 to 0.1) | -0.18 (-0.39 to 0.03) | -0.71 (-0.94 to -0.48) |
| **Gynoid fat mass**, kg | 0.47 (0.2 to 0.74) | -0.17 (-0.5 to 0.16) | -0.32 (-0.6 to -0.04) | -0.96 (-1.27 to -0.64) |
| Per-protocol analysis. The results are adjusted for age group (</≥ 40 years) and sex. Changes are estimated mean difference (95% confidence intervals) within-group. ^a^Outcomes previously reported (1). | | | | |

## Figures

**Figure S1: CONSORT flow diagram**


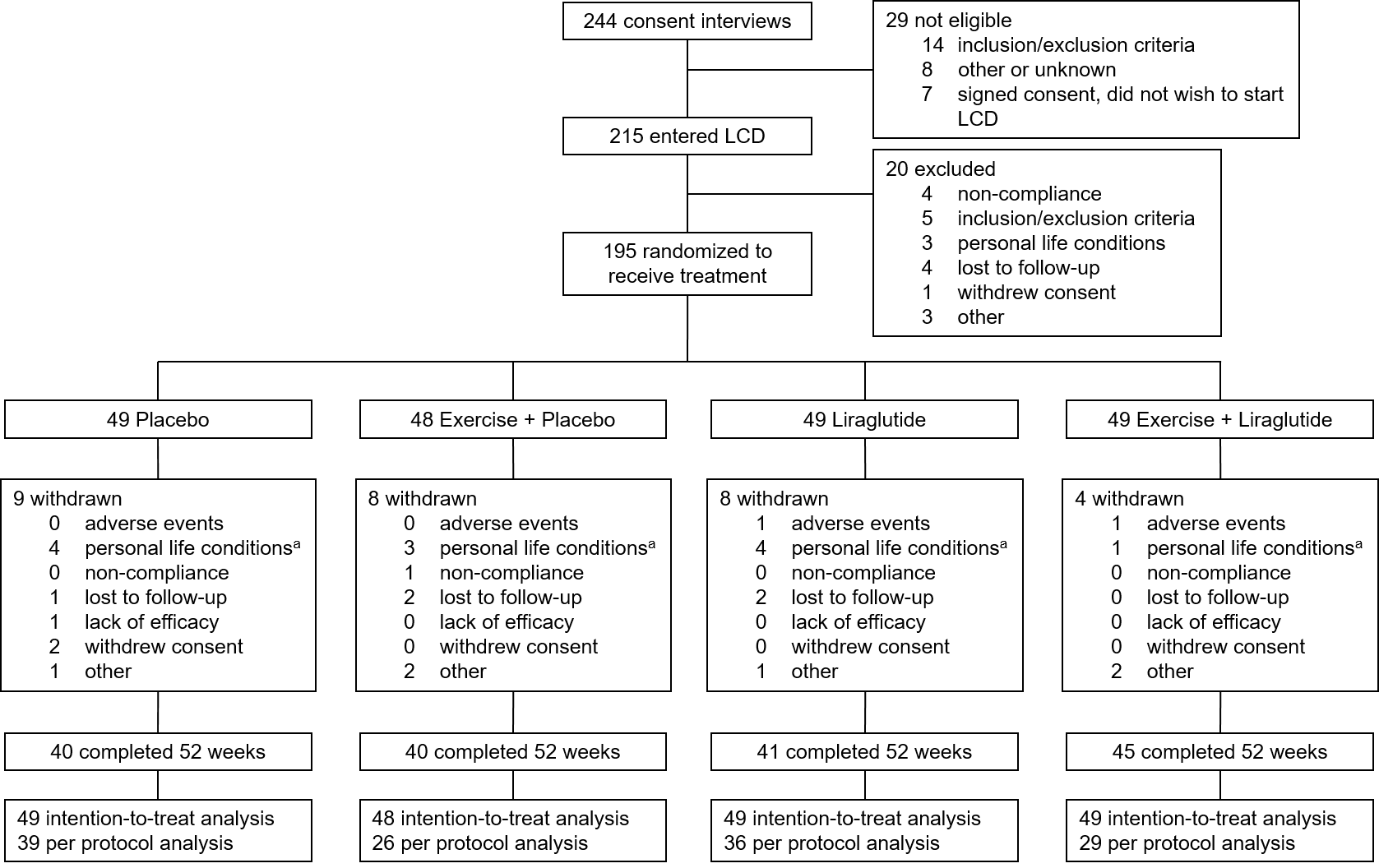


CONSORT diagram of the participants enrolled. ^a^personal life conditions: job-related, moving, disease, or death in the family. LCD: low-calorie diet.

**Figure S2: Observed MetS-Z of Women by Randomization Group**

**
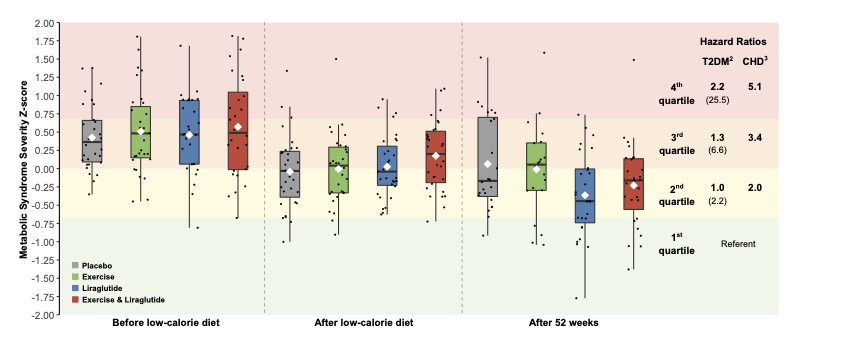
**

**Figure S3: Observed MetS-Z of Men by Randomization Group**

**
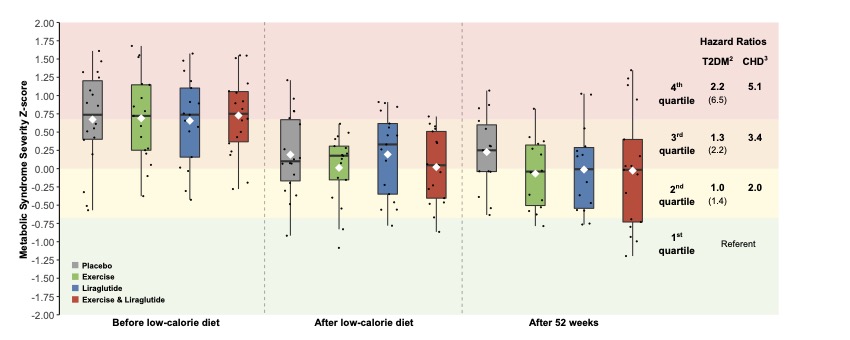
**

Observed MetS-Z of individual per-protocol participants (black dots) by randomization group at the three visits, before the low-calorie diet (week -8), after the low-calorie diet (week 0, randomization), and end of trial (week 52), presented as box plots. Tops of the boxes indicate the upper quartile; bottom of the box is the lower quartile; white diamonds observed mean; black horizontal line medians; whiskers ±1.5 times the interquartile range or highest or smallest observation. Box plots overlay MetS-Z quartiles associated with risk of future diabetes (2) and coronary heart disease (3) compared to the first quartile for white women/men and adjusted for individual MetS factors. For diabetes, unadjusted risks are also shown in parentheses. Number of women/men in the placebo group: 24/15, exercise group: 15/11, liraglutide group: 23/13, combination group: 18/11 after the low-calorie diet. MetS-Z: metabolic syndrome severity z-score. T2DM: type 2 diabetes, CHD: coronary heart disease.

## Methods

###

### Metabolic Syndrome Severity Z-score Factor Loadings & Formula (4)

Male non-Hispanic white

$\text{MetS-Z} = -5.4559 + 0.0125 \cdot WC - 0.0251 \cdot HDL + 0.0047 \cdot SBP + 0.8244 \cdot ln(TRI) + 0.0106 \cdot GLU$

Female non-Hispanic white

$\text{MetS-Z} = -7.2591 + 0.0254 \cdot WC - 0.0120 \cdot HDL + 0.0075 \cdot SBP + 0.5800 \cdot ln(TRI)+ 0.0203 \cdot GLU$

WC: waist circumference, HDL: high-density lipoprotein cholesterol, SBP: systolic blood pressure, TRI: triglycerides, GLU: fasting blood glucose

### Homeostatic model assessment of insulin resistance (HOMA-IR)

Fasting insulin levels (milliunits/mL) multiplied with the fasting glucose level (mmol/liter), divided by 22.5. Conversion factors: Insulin: 1 μU per mL equal to 6.00 pmol/L; Glucose: 1 mg/dL equal to 0.05551 mmol/L (1).

### Dual-Energy X-Ray Absorptiometry: Android & Gynoid Region of Interest

The android region of interest was defined by the software as the area of the trunk limited by two horizontal lines; the pelvic line and a line placed 20% cranially of the length between the pelvic line and neck cut line. The upper gynoid line was defined as 1.5 times the length of the android region and placed inferior to the pelvic line. The lower gynoid line was placed twice the height of the android region from the upper gynoid line (5).

## References

1. Lundgren JR, Janus C, Jensen SBK et al. Healthy Weight Loss Maintenance with Exercise, Liraglutide, or Both Combined. New England Journal of Medicine 2021;384:1719-1730.

2. Gurka MJ, Golden SH, Musani SK et al. Independent associations between a metabolic syndrome severity score and future diabetes by sex and race: the Atherosclerosis Risk In Communities Study and Jackson Heart Study. Diabetologia 2017;60:1261-1270.

3. Deboer MD, Gurka MJ, Golden SH et al. Independent Associations Between Metabolic Syndrome Severity and Future Coronary Heart Disease by Sex and Race. Journal of the American College of Cardiology 2017;69:1204-1205.

4. Gurka MJ, Lilly CL, Oliver MN, Deboer MD. An examination of sex and racial/ethnic differences in the metabolic syndrome among adults: A confirmatory factor analysis and a resulting continuous severity score. Metabolism 2014;63:218-225.

5. Bouchi R, Nakano Y, Ohara N et al. Clinical relevance of dual-energy X-ray absorptiometry (DXA) as a simultaneous evaluation of fatty liver disease and atherosclerosis in patients with type 2 diabetes. Cardiovasc Diabetol 2016;15:64.
